# Supplementary material for: Behavioral health and experience of violence among cisgender heterosexual and lesbian, gay, bisexual, transgender, queer and questioning, and asexual (LGBTQA+) adolescents in Thailand
Source: PLoS One. 2023 Jun 15;18(6):e0287130. doi: 10.1371/journal.pone.0287130 (PMC10270608; doi:10.1371/journal.pone.0287130)
Supplement: S4 File — Partial English-language translation of the study questionnaire. (DOCX) [file pone.0287130.s004.docx]

| **(For staff)** | **R** ⬜  **P** ⬜ ⬜ **SC** ⬜  **CR** ⬜ **/** ⬜ ⬜  **ST** ⬜ ⬜ | | | | | | |
| --- | --- | --- | --- | --- | --- | --- | --- |
|  | area | ⬜1) Urban | | ⬜2) Rural | | | |
|  | type | ⬜1) Public | | ⬜2) Private | | | |
|  | grp | ⬜1) General education | | ⬜2) Vocational | | | |
|  | level | 1) Matthayom 1  3) Matthayom 5 | | χ2) Matthayom 3  4) Vocational Education 2 | | | |
| **The 5th National School Survey on Alcohol Consumption, Substance Use and Other Health-Risk Behaviors, Academic Year 2020** | | | | | | | |
| **Section A. General Characteristic of the Student** | | | | | | | |
| **Instructions:** Please mark 🗸 in the box □ or fill in the blank...............**without writing your name or student identification number** | | | | | | | |
| 1. *Sex at Birth* | | ⬜1) Male (*Dek Chai / Nai*) ⬜ 2) Female (*Dek Ying / Nang Sao / Nang*) | | | | | |
| 1b. *Gender Identity (with what gender do you actually identify?)* | | ⬜1) Male ⬜ 2) Female ⬜ 3) Gender diverse  ⬜4) Not sure ⬜ 9) Refuse to answer | | | | | |
| 2. Age | | ⬜ ⬜ Years. Born in Year B.E. ⬜ ⬜ ⬜ ⬜ | | | | | |
| 3. Place of birth | | Province................................... | | | | | |
| 4. Religion | | ⬜1) Buddhism ⬜2) Islam  ⬜3) Christianity ⬜4) Others, specify................................... | | | | | |
| 5. *Currently, where do you stay?* | | ⬜ 1) Family house / flat ⬜ 2) School dormitory  ⬜ 3) Dormitory / rented home / apartment, non-family  ⬜ 4) Other, please specify.................. | | | | | |
| 6. *Currently,* with whom do you stay? (Multiple answers allowed) | | ⬜1) Alone ⬜2) Parents ⬜3) Father ⬜4) Mother ⬜5) Relatives ⬜6) Friend ⬜7) Romantic partner ⬜8) Other, please specify.......................................... | | | | | |
| 8. What was your grade point average in the previous semester? | | ⬜ . ⬜ ⬜ | | | | | |
| 9. How are you grades this school year compared to the previous one? | | ⬜1) This year is the same  ⬜2) This year’s grades are better  ⬜3) This year’s grades are worse | | | | | |
| 10. How much money do you receive as allowance each week? | | ...............................................Bahts | | | | | |
| 11. In the past 12 months, have you worked to earn an income? | | ⬜ 0) No | | | | ⬜1) Yes | |
| 12. In the past 12 months, have you ever been suspended from school due to disciplinary reason? | | ⬜ 0) No | | | | ⬜1) Yes | |
| 13. Do you have the tendency to leave school before finishing *Matthayom 6* / *Por Wor Chor 3* (12th Grade)? | | ⬜ 0) No | | | | ⬜1) Yes | |
| 14. During the past semester, how often do you play sports / exercise each week? | | ⬜0) None  ⬜2) 3-4 days per week | | | ⬜1) 1-2 days per week  ⬜3) 5-7 days per week | | |
| 15. During the past semester, on school days, how many hours of sleep did you get on average? | | ⬜1) 4 hours or less | ⬜2) 5 hours | | | | ⬜3) 6 hours |
|  |  | ⬜4) 7 hours | ⬜5) 8 hours | | | | ⬜6) 9 hours |
|  |  | ⬜7) 10 hours or more | | | | | |

| **Section D. Tobacco Use** | |
| --- | --- |
| **Instructions:** Please mark 🗸 in the box □ or fill in the blank............... | |
| 1. In your lifetime, have you smoked more than 5 packs or 100 sticks of cigarettes? | ⬜ 0) Never smoked **(*skip to Question 10*)**  ⬜ 1) Yes, but not more than 5 packs or 100 sticks  ⬜ 2) Yes |
| 1. How old were you when you ***started*** smoking entire cigarettes or other tobacco products | Started smoking at...............years of age |
| 1. When was the most recent occasion when you smoked cigarette? | ⬜ 1) More than 12 months ago **(*skip to Question 10*)**  ⬜ 2) More than 30 days ago but within past 12 months  ⬜ 3) More than 1 week ago but within past 30 days  ⬜ 4) Within the past 1 week |
| 4. On the most recent occasion when you smoked, how did you obtain the cigarette?  **(choose only one answer)** | ⬜ 1) Purchased by self (from store, stall / online shop)  ***(If purchased by self with no other response, answer the next question; If other responses were also selected, go to Question 5)***  ⬜ 2) Nominee purchase (buyer was more than 20 years of age)  ⬜ 3) Nominee purchase (buyer was less than 20 years of age)  ⬜ 4) Given by another person (who was more than 20 years of age)  ⬜ 5) Given by another person (who was less than 20 years of age)  ⬜ 6) Nominee purchase  ⬜ 7) Stolen  ⬜ 8) Another method |
| 4b. If purchased by self, did the vendor check your ID? | ⬜ 0) No ***(skip to Question 4d)***  ⬜ 1) Yes |
| 4c. Whose identification card did you use to make the purchase? | ⬜ 1) Own card  ⬜ 2) Another person’s card  ⬜ 3) Used a fake ID  ⬜ 9) Refuse to answer |
| 4d. Did you bribe the seller? | ⬜ 0) Did not bribe  ⬜ 1) Bribed  ⬜ 9) Refuse to answer |
| 5. If you wanted to smoke a cigarette, how long would it take you to buy/find one? | It would take....................... minutes |
| 6. In the past 12 months, have you ever tried to quit smoking cigarettes? | ⬜0) No  ⬜1) Yes |
| 7. In the past 12 months, have you ever stopped smoking cigarettes? | ⬜0) No  ⬜1) Yes, for ………….. days |
| 8. In the past 30 days, how many days did you smoke? | ⬜ 0) None ***(Skip to Question 10)***  ⬜ 1) 1-2 days  ⬜ 2) 3-5 days  ⬜ 3) 6-9 days  ⬜ 4) 10-19 days  ⬜ 5) 20 days or more |
| 9. In the past 30 days, on the days that you smoked, how many cigarettes did you use? |  1) Less than 1 per day   2) 1 per day   3) 2-5 per day   4) 6-10 per day   5) 11-20 per day   6) More than 20 per day |
| 10. In your lifetime, have you ever used an electronic cigarette? | ⬜ 0) Never smoked **(*skip to Section E*)**  ⬜ 1) Yes  a) I started when I was ……………. years old |
| 11. When was the most recent occasion when you used an electronic cigarette? | ⬜ 1) More than 12 months ago **(*skip to Section E1*)**  ⬜ 2) More than 30 days ago but within past 12 months  ⬜ 3) More than 1 week ago but within past 30 days  ⬜ 4) Within the past 1 week |
| 12. On the most recent occasion when you used, how did you obtain the electronic cigarette? | ⬜ 1) Purchased by self (from store, stall / online shop)  ***(If purchased by self, answer the next question; If another response was selected, go to Question 13)***  ⬜ 2) Nominee purchase (buyer was more than 20 years of age)  ⬜ 3) Nominee purchase (buyer was less than 20 years of age)  ⬜ 4) Given by another person (who was more than 20 years of age)  ⬜ 5) Given by another person (who was less than 20 years of age)  ⬜ 6) Nominee purchase  ⬜ 7) Stolen  ⬜ 8) Another method |
| 13. In the past 12 months, have you ever tried to quit using electronic cigarette? | ⬜0) No  ⬜1) Yes |
| 14. In the past 12 months, have you ever stopped using electronic cigarette? | ⬜0) No  ⬜1) Yes, for ………….. days |
| 15. In the past 30 days, how many days did you use electronic cigarette? | ⬜ 0) None ***(Skip to Section E)***  ⬜ 1) 1-2 days  ⬜ 2) 3-5 days  ⬜ 3) 6-9 days  ⬜ 4) 10-19 days  ⬜ 5) 20 days or more |
| 16. In the past 30 days, on the days that you used electronic cigarette, how many times per day did you use electronic cigarette? cigarettes did you use? (one "TIME" consists of around 15 puffs or lasts around 10 minutes) |  1) Less than 1 per day   2) 1 per day   3) 2-5 per day   4) 6-10 per day   5) 11-20 per day   6) More than 20 per day |

| **Section E. Alcohol Use** | |
| --- | --- |
| **E1 Drinking behavior** | |
| 1. In your lifetime, have you ever drank alcohol (not including sipping or tasting in very small amount)? | ⬜ 0) Never drank ***(skip to Section E3)*** |
|  | ⬜ 1) Yes |
| 2. When did you **start** drinking alcohol for the first time? | Started drinking at age...............years |
| 3. When was the most recent occasion that you drank (not including sipping or tasting in very small amount)?) | ⬜ 1) More than 12 months ago  ⬜ 2) More than 30 days ago but within past 12 months  ⬜ 3) More than 1 week ago but within past 30 days  ⬜ 4) Within the past 1 week |
| 4. On the most recent occasion when you drank, how did you obtain alcohol?  **(choose only one answer)** | ⬜ 1) Purchased by self (from store, stall / online shop)  ***(If purchased by self, answer the next question; If another response was selected, go to Question 5)***  ⬜ 2) Nominee purchase (buyer was more than 20 years of age)  ⬜ 3) Nominee purchase (buyer was less than 20 years of age)  ⬜ 4) Given by another person (who was more than 20 years of age)  ⬜ 5) Given by another person (who was less than 20 years of age)  ⬜ 6) Nominee purchase  ⬜ 7) Stolen  ⬜ 8) Another method |
| 4a. If purchased by self, did the vendor check your ID? | ⬜ 0) No ***(skip to Question 4c)***  ⬜ 1) Yes |
| 4b. Whose identification card did you use to make the purchase? | ⬜ 1) Own card  ⬜ 2) Another person’s card  ⬜ 3) Used a fake ID  ⬜ 9) Refuse to answer |
| 4c. Did you bribe the seller? | ⬜ 0) Did not bribe  ⬜ 1) Bribed  ⬜ 9) Refuse to answer |
| 5. If you wanted to drink alcohol, how long would it take you to buy/find a drink? | It would take....................... minutes |
| 6. **In the past 30 days,** how many times did you drink? | ⬜ 0) None ***(Skip to Section E3)***  ⬜ 1) 1-2 times  ⬜ 2) 3-5 times  ⬜ 3) 6-9 times  ⬜ 4) 10-19 times  ⬜ 5) 20 times or more |

| **1 unit of alcohol** or 1 drink **=** 1 glass of beer, or 1 glass of wine, or 1 shot of whiskey / brandy  [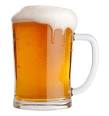](http://www.google.co.th/url?url=http://www.cutykiss.com/store/article/view/%E0%B8%84%E0%B8%A7%E0%B8%B2%E0%B8%A1%E0%B8%94%E0%B8%B1%E0%B8%99%E0%B8%95%E0%B9%88%E0%B8%B3_%E0%B8%81%E0%B8%B4%E0%B8%99%E0%B9%80%E0%B8%9A%E0%B8%B5%E0%B8%A2%E0%B8%A3%E0%B9%8C%E0%B8%8A%E0%B9%88%E0%B8%A7%E0%B8%A2%E0%B9%84%E0%B8%94%E0%B9%89%E0%B8%88%E0%B8%A3%E0%B8%B4%E0%B8%87%E0%B8%AB%E0%B8%A3%E0%B8%B7%E0%B8%AD-121711-th.html&rct=j&frm=1&q=&esrc=s&sa=U&ei=vnWGVdSZLo-7uATvpYKgDA&ved=0CBsQ9QEwAw&sig2=L8DqNwsj3qelAZDa-vCtMw&usg=AFQjCNHf_5KYD6BUZ2A32hJrQjd5IS_TIw)  1 cup (50 ml.)  beer 1 glass (280-285 ml.)  wine 1 glass (100 ml.)  Whiskey 1 shot (30 ml.) or 1 large cap | | | | | |
| --- | --- | --- | --- | --- | --- |
| 7. **In the past 30 days**, on each occasion that you drink, how many drinks do you usually take? | ⬜0) Never  ⬜3) 3 drinks  ⬜6) 6 drinks | ⬜1) 1 drink  ⬜4) 4 drinks  ⬜7) 7 drinks | | | ⬜2) 2 drinks  ⬜5) 5 drinks  ⬜8) 8 drinks |
|  | ⬜9) 9 drinks | ⬜10) 10 drinks or more | | | |
| 8. **In the past 30 days**, how many times did you take 5 drinks or more on the same occasion? | ⬜0) Never  ⬜3) 3-5 times | ⬜1) 1 time  ⬜4) 6-9 times | | | ⬜2) 2 times  ⬜5) 10 times or more |
| 9. **In the past 30 days**, how many times did you drink alcohol to the point of drunkenness? | ⬜0) Never  ⬜3) 6-9 times | ⬜1) 1-2 times  ⬜4) 10-19 times | | | ⬜2) 3-5 times  ⬜5) 20 times or more |
| 10. When you drink, what was the **most common** drinking spot? (choose only one answer) | ⬜1) School / school event | | | ⬜2) Dormitory | |
|  | ⬜3) Own or friend’s home | | | ⬜4) Farm / orchard | |
|  | ⬜5) Restaurant, club, or bar **no more than** 300 m. from school  ⬜6) Restaurant, club, or bar **more than** 300 m. from school  ⬜7) Others, specify............................ | | | | |
| 11. Approximately how much do you spend on drinking each month? | ⬜0) Did not pay | | ⬜1) Paid …………..………..Bahts | | |

| **Section F. Use of Drugs or Substances** | | | | | | | |
| --- | --- | --- | --- | --- | --- | --- | --- |
| **Instructions** Please mark 🗸 **in the blank space that corresponds to your answer** (if you do not wish to answer, please leave the cell blank) | | | | | | | |
| **F1. Have you ever used the following drug or substance?** | **1) In your lifetime (if “No”, go to the next row)** | | **2) In the past 12 months (if “No”, go to the next row)** | | **3) In the past 30 days วัน** | |  |
|  | **No** | **Yes** | **No** | **Yes** | **No** | **Yes** |  |
| a. Analgesic (not as medication) |  |  |  |  |  |  |  |
| b. Antihistamine (not as medication) |  |  |  |  |  |  |  |
| c. Cough syrup (not as medication) |  |  |  |  |  |  |  |
| d. Anxiolytics |  |  |  |  |  |  |  |
| e. Sedatives |  |  |  |  |  |  |  |
| f. Energy drinks |  |  |  |  |  |  |  |
| g. *PRO* [procodyl, promethazine] |  |  |  |  |  |  |  |
| h. *LEAN* [purple drank] |  |  |  |  |  |  |  |
| i. Poppers |  |  |  |  |  |  |  |
| j. Kratom leaf |  |  |  |  |  |  |  |
| k. Kratom leaf tea mixed with other substance (4x100) |  |  |  |  |  |  |  |
| l. Cannabis |  |  |  |  |  |  |  |
| m. Opium |  |  |  |  |  |  |  |
| n. Ecstasy / Love Drug |  |  |  |  |  |  |  |
| o. Ketamine |  |  |  |  |  |  |  |
| p. Heroin |  |  |  |  |  |  |  |
| q. Inhalants (paint thinner, glue, benzene) |  |  |  |  |  |  |  |
| r. *Ya ba* (methamphetamine) |  |  |  |  |  |  |  |
| s. *Ice* (crystal methamphetamine) |  |  |  |  |  |  |  |

**Note:** If you have **never used any substance** (answer “No” on all items in the sub-section 1 “**Lifetime**”), please **skip to Section G (Awareness and Attitude toward Drinking and Drug Use)**

If you **have used any substance** above, please **answer the following questions F2 thru F4** before going to **Section G**.

| **Section H. Sexual Activity** | | | | | | | | | | |
| --- | --- | --- | --- | --- | --- | --- | --- | --- | --- | --- |
| **Instructions:** Please mark 🗸 in the box □ or fill in the blank............... | | | | | | | | | | |
| 1a. Have you ever had a romantic partner (*fan*) | | | | ⬜ 0) No  ⬜ 1) Yes  ⬜ 9) Refuse to answer | | | | | | |
| 1b. *To which gender are you attracted? (multiple answers allowed)* | | | |  1) Male   2) Female   3) Transgender female / kathoey   4) Transgender male / tom   5) Neither male nor female   6) I'm not sure to whom I am attracted   7) I'm not attracted to any gender   9) Refuse to answer | | | | | | |
| 1c. *Have you ever had sex? (Not including manual, oral, or object-based contacts)* | | | | ⬜ 0) Never ***(skip to Section I)***  ⬜ 1) Yes a) I had sex for the first time when I was …........... years old  ⬜ 9) Refuse to answer ***(skip to Section I)*** | | | | | | |
| 2. **In your lifetime**, with how many partners have you had sex? | | | | ⬜1) 1 person | | ⬜2) 2 persons | | | ⬜3) 3 persons | ⬜4) 4 persons |
|  |  |  |  | ⬜5) 5 persons | | ⬜6) 6 persons | | | ⬜7) More than 6 persons | |
| 2b. **In the past 12 months**, with how many partners have you had sex? | | | | ⬜1) 1 person ⬜2) 2 persons ⬜3) 3 persons ⬜4) 4 persons  ⬜5) 5 persons ⬜6) 6 persons ⬜7) More than 6 persons | | | | | | |
| 3. **On the latest occasion,** did you drink alcohol before or during sex? | | | | ⬜1) No ⬜2) Yes ⬜9) Refuse to answer | | | | | | |
| 4. **On the latest occasion,** did you use substances before or during sex? | | | | ⬜1) No ⬜2) Yes ⬜9) Refuse to answer | | | | | | |
| 5. **On the last occasion that you had sex**, what birth control did you and your partner use? (More than 1 answer allowed) | | | | | | | | | | |
| ⬜ 0) None | ⬜ 1) External ejaculation | | | ⬜ 2) Wore condom | | | | ⬜ 3) Birth control patch | | |
| ⬜ 4) Birth control pill | ⬜ 5) Emergency contraceptive pill | | | ⬜ 6) Contraceptive injection | | | | ⬜ 7) Contraceptive implant | | |
| ⬜ 8) Suppository | ⬜ 9) Counting days | | | ⬜ 10) Other methods, specify........................ | | | | | | |
| 6. How many times have you been pregnant or impregnated someone? | | | | ⬜ 0) Never (Skip to Question 8) | | | | | ⬜1) 1 Time | |
|  |  |  |  | ⬜ 2) 2 Times or more | | | | | ⬜3) Not sure | |
| 7. If you found out that you or your partner were pregnant, what would you do? (More than 1 answers allowed) | | | | | | | | | | |
| ⬜1) Carry out the pregnancy and care for the baby to the best of my ability | | | | | ⬜ 2) Try to resolve the issue by myself without telling anyone | | | | | |
| ⬜ 3) Consult partner | | ⬜ 4) Consult teacher | ⬜ 5) Consult own parents | | | | ⬜ 6) Consult partner’s parents | | | |
| ⬜ 7) Consult friend / acquaintance | | | ⬜ 8) Terminate the pregnancy (abortion) | | | | | | | |
| ⬜ 9) Consult relevant organizations e.g., health center, hospital, consultation clinic  ⬜ 10) Other, please specify ……………………. | | | | | | | | | | |
| 8. Have you ever been taught about AIDS or HIV at school? | | | | ⬜ 0) Never  ⬜ 2) Not sure | | | | | ⬜ 1) Yes | |
| 9. Have you ever been taught about birth control at school? | | | | ⬜ 0) Never ⬜ 1) Yes  ⬜ 2) Not sure | | | | | | |

| **Section I. Safety and Violence** | | | | | | | | | |
| --- | --- | --- | --- | --- | --- | --- | --- | --- | --- |
| **Instructions:** Please mark 🗸 in the box □ with regard to your behaviors in the past 12 months and 30 days | | | | | | | | | |
| How often did you engage in the following behaviors/activities? | **In the past 12 months** | | | **In the past 30 days** | | | | | |
|  | Did not ride or sit in such vehicles | No | Yes | Did not ride or sit in such vehicles | Never | Occasionally | | Often | Always |
| 1. Wear a safety helmet when you ride a motorcycle or sit on the back pillion |  |  |  |  |  |  | |  |  |
| 2. Fasten the safety belt when driving a car or sitting in the passenger seat |  |  |  |  |  |  | |  |  |
| 3. Drive a car, ride a motorcycle or other vehicles immediately after drinking |  |  |  |  |  |  | |  |  |
| 4. Carry a knife, firearm, bat, or other objects to use as a weapon |  |  |  |  |  |  | |  |  |
| 5. Be threatened or assaulted with a weapon, e.g., a knife, firearm, bat, or another weapon |  |  |  |  |  |  | |  |  |
| 6. Get into a fight or a physical altercation with another person without being injured |  |  |  |  |  |  | |  |  |
| 7. Get into a fight or a physical altercation with another person to the point where you are injured and require medical treatment |  |  |  |  |  |  | |  |  |
| 8. In the past 12 months, has your romantic partner ("fan") ever hit or intentionally assaulted you? | | | |  0) No   1) Yes   2) Never had a romantic partner | | | | | |
| 9. In the past 12 months, have you ever been forced to have sex without consent? | | | |  0) No   1) Yes   2) Never had sex | | | | | |
| 10. In the past 12 months, have you ever felt down, depressed, or hopeless in your life nearly every day for 2 consecutive weeks, to the point where you could not carry out daily routines as normal? | | | |  0) No   1) Yes | | | | | |
| 11. In the past 12 months, have you ever seriously considered committing suicide? | | | | ⬜ 0) No | | | ⬜1) Yes | | |
| 12. In the past 12 months, have you ever made plans for suicide? | | | | ⬜ 0) No | | | ⬜1) Yes | | |
| 13. In the past 12 months, how many times have you actually attempted suicide? | | | | ⬜ 0) None  ⬜1) Yes, ............... time(s) | | | | | |
| 14. In the past two weeks, how often have you felt little interest or pleasure in doing things? | | | | 0) Not at all  1) Some days  χ2) Often, more than 7 days  3) Every day or nearly every day | | | | | |
| 15. In the past two weeks, how often have you felt down, depressed, or hopeless? | | | | 0) Not at all  1) Some days  χ2) Often, more than 7 days  3) Every day or nearly every day | | | | | |
