# Supplementary material for: Behavioral health and experience of violence among cisgender heterosexual and lesbian, gay, bisexual, transgender, queer and questioning, and asexual (LGBTQA+) adolescents in Thailand
Source: PLoS One. 2023 Jun 15;18(6):e0287130. doi: 10.1371/journal.pone.0287130 (PMC10270608; doi:10.1371/journal.pone.0287130)
Supplement: S5 File — Characteristics of study participants who provided complete vs. incomplete information regarding gender and sexuality. (DOCX) [file pone.0287130.s005.docx]

# **Supplementary Table 1. Characteristics of study participants who provided complete vs. incomplete information regarding gender and sexuality, weighted percent ± SE unless otherwise noted (n= 23,659 participants) (COLUMN PERCENTS)**

| **Characteristic** | **Provided complete information** | **Incomplete information** | **P-value** |
| --- | --- | --- | --- |
|  | **(n=21,323)** | **(n=2,336)** |  |
| **School type** |  |  |  |
| Government | 72.9% ± 2.0% | 77.0% ± 2.2% | 0.1262 |
| Private | 27.1% ± 2.0% | 23.0% ± 2.2% |  |
| **Year Level** |  |  |  |
| Mathayom 1 (Year 7) | 35.8% ± 3.7% | 30.7% ± 5.6% | 0.2234 |
| Mathayom 3 (Year 9) | 36.0% ± 3.7% | 31.4% ± 6.1% |  |
| Mathayom 5 (Year 11, General Education) | 25.3% ± 7.0% | 33.9% ± 10.9% |  |
| Vocational Certificate 2 (Year 11, Vocational Education) | 2.9% ± 1.1% | 4.0% ± 2.0% |  |
| **Religion** |  |  |  |
| Buddhism | 86.8% ± 3.2% | 90.5% ± 2.4% | **0.040** |
| Islam | 9.8% ± 3.3% | 6.3% ± 2.2% |  |
| Christianity | 2.7% ± 0.9% | 2.2% ± 0.6% |  |
| Others | 0.8% ± 0.1% | 1.1% ± 0.3% |  |
| **Region** |  |  |  |
| Special-Bangkok | 8.9% ± 6.5% | 5.7% ± 4.3% | **<0.001** |
| Bangkok Metro Areas | 11.3% ± 7.9% | 8.4% ± 6.1% |  |
| Central | 20.3% ± 8.5% | 19.0% ± 8.1% |  |
| South | 22.3% ± 8.7% | 16.4% ± 6.4% |  |
| North | 22.5% ± 7.9% | 17.5% ± 6.4% |  |
| Northeast | 14.8% ± 5.6% | 32.9% ± 11.3% |  |
| **Living Situation**** |  |  |  |
| Family house/flat | 87.1% ± 1.7% | 91.1% ± 1.8% | **0.021** |
| School dorm | 3.1% ± 1.0% | 2.4% ± 1.0% |  |
| Outside dorm | 9.8% ± 1.4% | 6.5% ± 1.4% |  |
| **Weekly allowance (THB) (mean** ± **standard errors)** | 480.7 ± 16.7 | 463.5 ± 23.1 | 0.305 |
| **Grade point average (GPA)** |  |  |  |
| GPA=0.1-1.0 | 0.1% ± 0.0% | 0.3% ± 0.2% | 0.232 |
| GPA=1.1-2.0 | 4.4% ± 0.4% | 4.5% ± 0.6% |  |
| GPA=2.1-3.0 | 29.9% ± 1.9% | 32.7% ± 2.6% |  |
| GPA=3.1-4.0 | 59.5% ± 1.5% | 56.9% ± 2.7% |  |
| Unknown | 6.1% ± 0.7% | 5.5% ± 1.2% |  |

Bold P-values denote statistical significance at 95% level of confidence
