# Supplementary material for: Behavioral health and experience of violence among cisgender heterosexual and lesbian, gay, bisexual, transgender, queer and questioning, and asexual (LGBTQA+) adolescents in Thailand
Source: PLoS One. 2023 Jun 15;18(6):e0287130. doi: 10.1371/journal.pone.0287130 (PMC10270608; doi:10.1371/journal.pone.0287130)
Supplement: Attachment — Submitted filename: Review.pdf [file pone.0287130.s006.pdf]

Disparities in Behavioral Health and Experience of Violence between Cisgender-Heterosexual vs. Lesbian, Gay, Bisexual, Transgender, Queer and Questioning, and Asexual (LGBTQA+) Thai Adolescents

Title, Abstract and Background

The title, abstract and introduction are adequate. The manuscript brings relevant information about the literature and clearly supports its justification and aims.

Methods:
The methods were also well written. I only have a few comments and suggestions for improving the manuscript.

Page 7. Line 105. "(…) am attracted"; "7) I'm not attracted to any gender", and; "9) Refuse to answer".

Comment: The answer options goes from 7 to 9. Is there an option 8 or it was it just a typing error?

Instrumentation: Measurement of Experience of Violence:

Page 8. Lines 144-146. "We excluded students who had never been in a relationship and students who had never had sex from analyses on intimate partner violence and sexual violence, respectively."

Suggestion: Excluding participants who reported that never had sex can be misleading. Those participants who answered that they never had sex do not necessarily have not suffered sexual violence, since they may have the understanding that an experience of sexual intercourse is always with consent (different from what happens in rape). So, if it is not feasible to change in the analyses, it would be important to insert this information in the discussion section.

Results:

Table 1. I suggest inserting sociodemographic characteristics of the sample in table 1, including information about mean age of the participants in each school year, race and family income.

Tables 2a and 2b. The row frequencies of the tables (2a and 2b) do not add up to 100%. Are the weighted percentages the responsible for that? If not, the authors should revise the tables.

Page 20. Lines 209-210. "With regards to mental health outcomes and health behaviors, prevalence of depression at time of study and within past year was higher among bisexual, transgender, asexual, and otherwise (…)".

Comment: This result should be presented with caution. In fact, the study did not assess the presence of depression. The purpose of the PHQ-2 is to screen for depression in a "first-step" approach. Then, the literature suggests that participants who screen positive (in PHQ-2) should be further evaluated with the PHQ-9 instrument to determine whether they meet criteria for a depressive disorder. So, the PHQ-2 is an instrument that could assess the presence of symptoms of depression. This information about the difference between presence of disorder and presence of symptoms is important and should be revised in all manuscript.

Suggestion: As the authors compared the continuous data, I suggest to insert information about the means and standard deviations of the groups in each comparison.

Table 4b. Part 2. Data on the asexual AFAB group should be reviewed. In table 4, the prevalence for asexual AFAB group is 0% and in the previous paragraph the authors say that "respondents who identified as asexual reported the lowest prevalence of all types of violence".

Discussion:

The discussion needs to be improved. The results presented have the potential to be discussed with the current literature on the subject, but the authors need to include more references and discuss with greater depth the studies presented.

Limitations are well described, but some appear mixed up throughout the discussion. I suggest that they should be entered under the "Strengths and Limitations" section. The same situation occurs with regard to suggestions that authors make about future studies, these information appear several times throughout the discussion. I suggest to add a paragraph with these suggestions in the limitation section or in the conclusion section.

The information about the possibility of recall bias when referring to the prevalence of depressive experiences in the last 12 months, could be added as a limitation in the "Strengths and Limitations" section.

Page 30. Lines 254-255. "Such prevalence is higher than a previously-estimated 8 percent prevalence [13], and is higher than the global average even when those who provided incomplete information were taken into account[14]

Suggestion 1: Please specify the global average of the cited study. Also, if possible, present studies from different countries.

Suggestion 2: It may be important to bring more information on the mental health of young people who identify as LGBTQIA+, for example, citing Meyer's minority stress theory. I also suggest discussing other studies on mental health, substance abuse, sexual behavior and exposure to violence.

Page 32. Lines 301-303. "The higher prevalence of drinking in certain groups could be a reflection of unhealthy coping mechanisms[26], or a reflection of broader societal trends [27]."

Suggestion: Please, insert the mentioned societal trends.

Page 32. Lines 303-306. "Such disparities were also observed for lifetime history of illicit drug use, although homosexual male participants seemed to have notably higher prevalence in nearly all types of substances compared to other groups, which differed from a similar study elsewhere [28]".

Suggestion: Please, give more information such as location and data about the cited study.
